# Induction of dysfunctional CD14^low^CD16^-^ monocytes in acute COVID-19 infection

**DOI:** 10.3389/fimmu.2026.1814210

**Published:** 2026-08-25

**Authors:** Salimeh Ebrahimnezhaddarzi, Aimee Altermatt, Hans Kek, Jingling Zhou, Irene Boo, James H. McMahon, Bradley J. Gardiner, Rachel Sacks-Davis, Heidi E. Drummer, Anthony Jaworowski, Anna C. Hearps

**Affiliations:** 1 Disease Elimination Program, Burnet Institute, Melbourne, VIC, Australia; 2 School of Health and Biomedical Sciences, RMIT University, Melbourne, VIC, Australia; 3 Department of Infectious Diseases, The Alfred Hospital and Monash University, Melbourne, VIC, Australia; 4 School of Population Health and Preventive Medicine, Monash University, Melbourne, VIC, Australia; 5 School of Population and Global Health, University of Melbourne, Melbourne, VIC, Australia; 6 Department of Microbiology and Immunology at The Peter Doherty Institute for Infection and Immunity, University of Melbourne, Melbourne, VIC, Australia; 7 Department of Microbiology, Monash University, Clayton, VIC, Australia; 8 Department of Infectious Diseases at The Peter Doherty Institute for Infection and Immunity, University of Melbourne, Melbourne, VIC, Australia

**Keywords:** CD14, COVID-19, inflammatory response, monocyte, monocyte subsets

## Abstract

**Introduction:**

Severe COVID-19 disease is characterised by a state of hyperinflammation. As key producers of inflammatory mediators in blood, altered inflammatory activity of monocytes within some individuals may contribute to adverse disease outcomes.

**Methods:**

Analysis of monocyte activity under settings of infection and inflammation can be challenged by activation-induced shedding of monocyte receptors traditionally used to identify monocyte subsets. Here, we utilised alternative, more robust immunophenotyping approaches to investigate the impact of COVID-19 infection on monocyte phenotype and inflammatory status.

**Results:**

Immunophenotype analysis of cryopreserved peripheral blood mononuclear cells from unvaccinated, previously COVID-19 naive individuals (median age 43 years, range 21-95, n=42) with a PCR-confirmed acute COVID-19 infection indicated expansion of a novel population of CD14_low_CD16- monocytes as compared to control individuals (adjusted p value <0.001) which was more pronounced in individuals with severe disease presentation (p=0.004 for mild vs severe disease). Expansion of this population during acute COVID-19 was confirmed using an alternative, CD14 and CD16-independent strategy for identifying monocyte subsets and was termed infection associated monocytes (IAM). IAM were refractory to ex vivo stimulation with lipopolysaccharide (LPS) and their proportion correlated with plasma levels of TNF and CXCL10 (p<0.05 for both). Monocyte subsets from individuals with acute COVID-19 exhibited reduced basal and LPS-stimulated production of inflammatory cytokines including IL-1β, TNF and IL-6, indicative of an inert monocyte state. Expansion of IAM and impaired inflammatory activity persisted for up to 3 months after acute COVID-19 infection.

**Discussion:**

COVID-19 is associated with expansion of a novel subset of monocytes with impaired inflammatory activity that persist for at least 3 months after acute infection. Whether this population is uniquely expanded by COVID-19, and the long term implications of its persistence post-acute disease, remain to be defined.

## Introduction

COVID-19 infection has a diverse presentation, ranging from asymptomatic infection to severe respiratory and systemic disease leading to death, yet the immunological mechanisms which underpin these divergent responses remain incompletely defined (1). In addition to acute respiratory distress, severe COVID-19 presentations are characterized by local and systemic hyper-inflammation. Myeloid cells, including blood monocytes and tissue macrophages, play a key role in initiating and regulating inflammatory responses in the body. Older individuals and those with chronic inflammatory comorbidities or infections have a heightened risk of adverse COVID-19 outcomes. These same populations also exhibit dysregulated monocyte activity including impaired phagocytosis, altered expression of activation and inhibitory receptors and expansion of inflammatory, CD16+ monocyte subsets (2–7). Characterizing how monocytes respond to and are impacted by acute infections may advance our understanding of the mechanisms that drive hyper-inflammatory, adverse responses to COVID-19 and other acute infections.

Blood monocytes are comprised of functionally distinct subsets, which are typically classified based on surface expression of CD14 and CD16 into classical (Class: CD14^+^CD16^-^; representing 85% of blood monocytes), intermediate (Int: CD14^+^CD16^+^) and non-classical (NC: CD14^-/low^CD16^+^) subsets (8–10). Class monocytes respond rapidly to infection through cytokine production and microbial killing by phagocytosis and serve as precursors to dendritic cells and tissue macrophages. A proportion of Class monocytes also differentiate into Int subsets, which possess high inflammatory and antigen presenting activity, and subsequently NC monocytes, which are the most mature subset responsible for patrolling the blood vessel endothelium (9) (11). Numerous studies have analyzed the phenotype of monocytes in acute COVID-19 infection and report substantial alterations to monocyte phenotype and subset proportion (12–21). Furthermore, there is evidence that COVID-19-induced changes to monocyte phenotype and function can persist for many months following acute infection (22) through processes including epigenetic and metabolic reprogramming reminiscent of trained immunity (23–26). These studies have almost exclusively focused on hospitalized individuals and/or those with severe presentations, with less information regarding the impacts of mild COVID-19. Furthermore, immunophenotypic identification of monocytes typically relies on surface expression of CD14 and CD16 receptors, which are highly prone to activation-induced enzymatic cleavage which precludes accurate evaluation in settings including acute infections. Alternative gating approaches for monocyte subset identification utilizing myeloid markers with more resilient expression has yielded unique insights into monocyte biology during viral infections (27), but have yet to be applied to the analysis of COVID-19.

In this study, we aimed to utilize novel monocyte immunophenotyping approaches better suited to pathogenic settings to interrogate the impact of acute and convalescent COVID-19 infection on monocytes in individuals of varying ages and disease severity.

## Methods

### Cohort description and sample processing

This study utilized cryopreserved plasma and peripheral blood mononuclear cells (PBMC) from individuals recruited following PCR-confirmed COVID-19 infection into the Alfred Health COVID-19 Biobank (Alfred Health Human Research and Ethics Committee Number 182/20) in Melbourne, Australia. Participants were enrolled between April-October 2020 where the circulating COVID-19 strains in Australia were early B.1 lineages (28) with no circulation of alpha, beta, delta or omicron variants during this period. Due to the scarcity of community COVID-19 transmission in Australia prior to this timepoint, participants were reasonably assumed to be experiencing their first COVID-19 infection, and all individuals were unvaccinated for COVID-19. Available samples were analyzed from COVID-19 cases at the following 3 timepoints (where available); during acute infection (<10 days post-diagnosis; median 4 days, IQR: 2–6 days, n=42) and at 1 month (median 31 days, IQR: 29-32, n=27) and 3 months (median 106 days, IQR: 99 – 112, n=18) after COVID-19 diagnosis. Cryopreserved samples from healthy controls of a comparable age and sex sampled prior to the COVID-19 pandemic were analyzed in parallel. Blood samples from COVID-19 cases and uninfected controls were both collected into EDTA anticoagulant, processed similarly prior to cryopreservation and were thawed and analyzed simultaneously for this study.

SARS-CoV-2 viral load was quantified by reverse transcription-quantitative PCR from cryopreserved aliquots of viral transport media containing nasal swab eluates collected from participants at the time of diagnosis as previously described (29).

### Plasma analyses

Plasma levels of C-reactive protein (CRP), soluble (s) CD14, C-C Motif Chemokine Ligand 2 (CCL2), sCD163, Tumor Necrosis Factor alpha (TNF) and C-X-C motif chemokine ligand 10 (CXCL10) were evaluated using a customized Magnetic Luminex Assay (R&D Systems, Minneapolis, Minnesota, USA). All samples were measured following manufacturer instructions and quantified on a Bio-Plex 200 System (Bio-Rad, Hercules, California, USA). Experimental data was analyzed by fitting a 5-parameter logistic curve for all measurements.

### Cellular immunophenotyping

PBMC were stained with LIVE/DEAD Yellow viability stain (Invitrogen, Carlsbad, CA) prior to surface immunophenotyping. *Ex vivo* inflammatory responses were evaluated following incubation of thawed PBMC in the absence or presence of 50ng/mL lipopolysaccharide (LPS, from *Escherichia coli* [O111:B4], Sigma-Aldrich, St Louis, MO) in Iscove’s Modified Dulbecco’s Medium supplemented with 100 units/mL penicillin, 100µg/mL streptomycin, 2mM L-glutamine (all from Gibco, ThermoFisher Scientific, Waltham, MA) and 10% human serum for 4 hours at 37 °C in a humidified incubator. These stimulation conditions have been demonstrated to be the most optimal for analysis of monocyte inflammatory responses (30). Brefeldin A (3µg/mL, eBiosciences, Waltham, MA) and GolgiStop™ (1:1500 final dilution, BD Biosciences) were added after 1 hour to prevent cytokine release. Intracellular expression of inflammatory cytokines in monocytes was subsequently detected following permeabilization with Perm/Wash buffer 1 (BD Biosciences). Details of all antibodies used in immunophenotyping analyses are described in **Supplementary Table 1**. Fluorescence minus one controls were used to define positive populations. Cells were acquired on a Fortessa LSR X20 flow cytometer (BD Biosciences) and data analyzed using FlowJo software (version 10, FlowJo LLC, Ashland, OR). Samples from cases and controls were treated and analyzed identically and simultaneously.

### Statistical analysis

Mann-Whitney U tests or Friedman test with Dunn’s multiple comparison test were performed to identify significant differences between groups or cell populations. Paired Wilcoxon signed rank analyses were used to compare different experimental conditions between matched samples. Chi-squared analyses were used for relevant demographic and clinical variable. To quantify COVID-19-related differences, multivariate linear regression, adjusted for age and sex, was used to compare immunological outcomes between controls and COVID-19+ individuals at acute infection, and one and three months post infection. The exposure was COVID-19 status (case=1, control=0). We reported the estimate and 95% confidence interval around the estimate for the mean difference in each immunological outcome value among cases and controls when sex and age were held constant. We fit one model for each outcome at each timepoint (**Supplementary Methods 1**).

We performed a sensitivity analysis to test the linearity assumption of linear regression, repeating the analysis using a log-transformation of skewed outcome variables. We found no difference in the interpretation of results so chose linear regression on untransformed outcomes for consistency. To adjust for multiple testing, all p-values derived from the regression analysis were adjusted using the Benjamini-Hochberg method (31) and adjusted p-values were reported.

To test for associations between proportions of CD14^low^CD16^-^ monocytes (identified using CD14 and CD16 gating) and soluble parameters/viral load among COVID-19+ individuals at acute infection, we used multivariate linear regression, adjusted for age and sex. The exposure was CD14^low^CD16^-^ value and the outcome was the soluble factor/viral load. We fit one model for each parameter and reported the estimate, 95% confidence interval and adjusted p-value from the linear regression. The results can be interpreted as the average increase in the parameter value associated with a 1% increase in CD14^low^CD16^-^ (**Supplementary Methods 2**).

## Results

### A novel population of CD14^low^CD16^-^ monocytes is observed in individuals with acute COVID-19 infection

The demographic characteristics of the study participants is given in **Table 1** and indicate a similar age and sex distribution between cases and controls. Of COVID-19+ individuals, 59.5% had mild infection and were not hospitalized, while the remainder were hospitalized either requiring (28.6%) or not requiring (11.9%) supplemental oxygen (full details of disease severity provided in **Table 1**). As expected, hospitalized individuals were on average significantly older than non-hospitalized individuals (**Table 1**) but nasopharyngeal viral load at diagnosis did not differ by disease status (data not shown).

**Table 1:** Clinical and demographic characteristic of the cohort.

Parameter	Controls	COVID-19	P value
n	40	42	
Median age (range)	40 (20-84)	43 (21-95)	0.584^1^
Female, n (%)	19 (47.5%)	22 (52.4%)	0.641^2^
COVID severity, n(%)			
- Non-hospitalized *Median age (range)*		25 (59.5%) 34 (20-84)	
- Hospitalized – not requiring O_2_ *Median age (range)*		5 (11.9%) 81 (40-92)	0.001^3^
- Hospitalized –requiring O_2_^4^ *Median age (range)*		12 (28.6%) 75 (34-95)	<0.001^3^

^1^ Significance assessed using Mann-Whitney U test.

^2^ Significance assessed using Chi squared test.

^3^ As compared to non-hospitalized using Mann-Whitney U test.

^4^ Includes supplemental oxygen (n=7), nasal high-flow oxygen and/or non-invasive mechanical ventilation (n=2) and invasive mechanical ventilation and/or ECMO (n=3).

**Figure 1:**
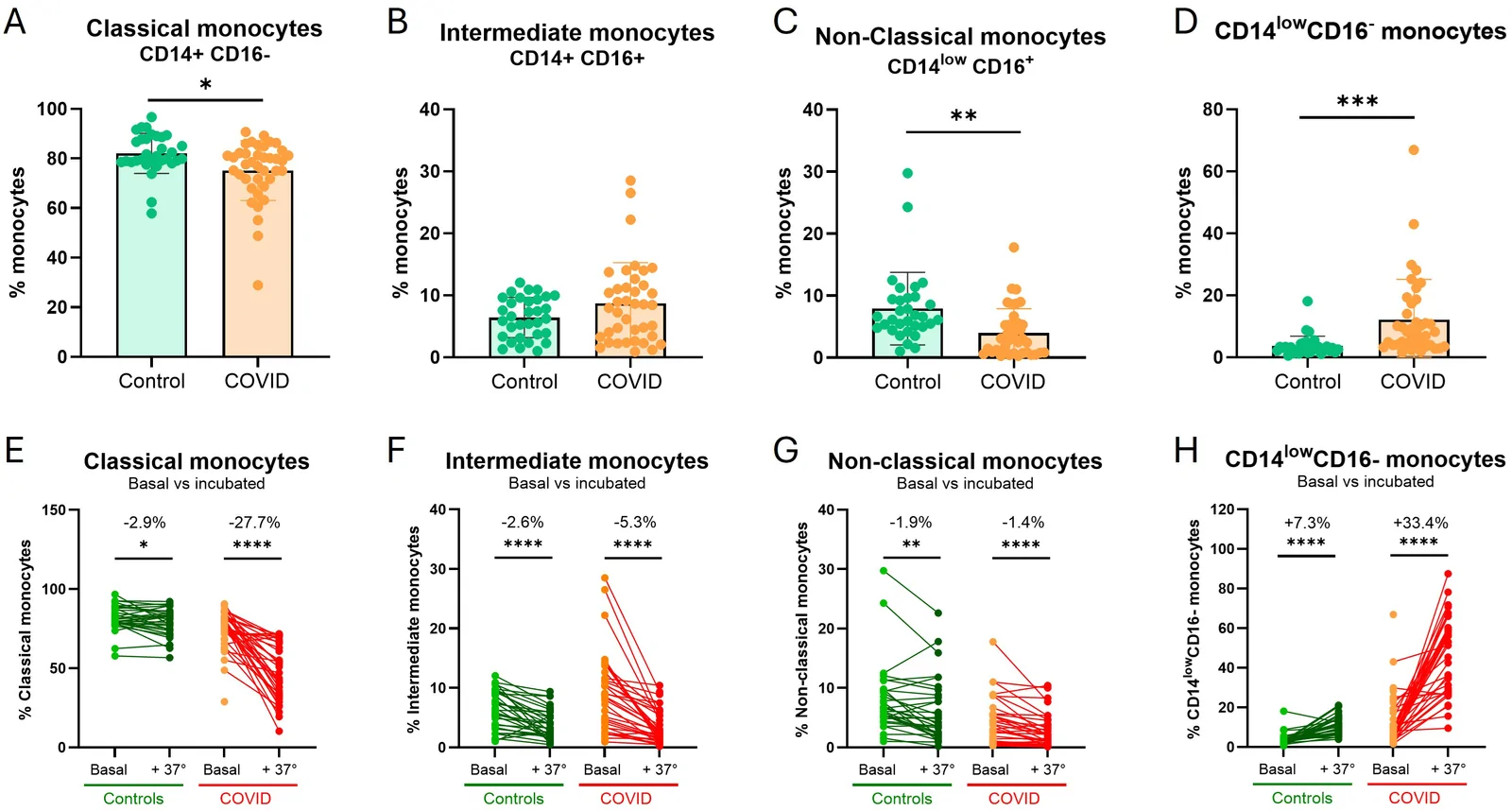
*Altered monocyte subset proportion and receptor instability in acute COVID infection.* **(A–D)** The proportion of classical [CD14+CD16-; **(A)**], intermediate [CD14+CD16+; **(B)**], non-classical [CD14^low^CD16+; **(C)**] and CD14^low^CD16^-^ **(D)** monocytes in uninfected controls (green bars) or COVID+ individuals during acute infection (orange bars) was evaluated in cryopreserved PBMC immediately following defrosting. **(E–H)** Monocyte subset proportion was assessed in PMBCs using CD14/CD16 gating from uninfected controls (green symbols) and those with acute COVID infection (orange/red symbols) either immediately after defrosting (Basal) or following culture at 37 °C for 4 hours (37°). Graphs show mean and standard error of the mean. *, **, and *** indicate p<0.05, <0.01 and <0.001 as determined by linear regression, adjusted for age and sex **(A–D)** or paired Wilcoxon signed rank test **(E–H)**.

To confirm previous observations regarding the impact of acute COVID-19 infection on monocyte subsets, we gated subsets based on CD14 and CD16 surface expression (see **Supplementary Figure 1**), and compared individuals with and without acute COVID-19 infection using regression analysis adjusted for age and sex given known impacts of age on monocyte subset distributions (2, 6). These analyses confirmed a significant decrease in proportions of classical (Class, adjusted p=0.019) and non-classical (NC, adjusted p=0.009) monocytes in acute COVID-19 infection while intermediate (Int) monocyte proportions were not significantly different (**Figure 1**, **Table 2**). This analysis also identified a population of monocytes with a CD14^low^CD16^-^ phenotype which was expanded in individuals with acute COVID-19 (adjusted p=0.001, **Figure 1D**, **Table 2**). Stratification of individuals by disease severity indicted that a significantly higher proportion of this novel CD14^low^CD16^-^ subset was observed in individuals with both mild (non-hospitalized) and more severe (hospitalized, p<0.001 for both as compared to controls) COVID-19 infection but was more pronounced in the latter (p=0.004 as compared to mild cases, **Supplementary Figure 2**). Comparison of young (<40 years) and older (>60 years) individuals indicated CD14^low^CD16^-^ monocyte subset expansion was observed in acute COVID-19 infection in both age groups (**Supplementary Figure 2**), which was also consistent with adjusted regression analysis indicating no significant effect of age on CD14^low^CD16^-^ proportion (not shown). Together, these data indicate a novel population of monocytes not captured by traditional CD14 and CD16 gating approaches is expanded during both mild and more severe acute COVID-19 infection.

**Table 2:** Differences in monocyte parameters between controls and acute COVID infection^1.^.

Monocyte subset:	Classical			Intermediate			Non-classical			CD14^low^CD16^-^/Infection-associated monocytes		
Parameter	D	95%	p (adjusted)	D	95%	p (adjusted)	D	95%	p (adjusted)	D	95%	p (adjusted)
Monocyte subsets analyzed immediately and gated using CD14 and CD16												
Monocyte subset %	**-6.1**	**(-10.3**, **-1.8**)	**0.019**	2.7	(0.14, 5.22)	0.091	**-3.7**	**(-6.0,-1.4)**	**0.009**	**7.1**	**(3.5, 10.6)**	**0.001**
Monocyte subsets gated using 5-receptor gating following incubation at 37 °C												
Monocyte subset % - Cultured	**-30.9**	**(-37.8, -24.0)**	**<0.001**	-0.2	(-1.5, 1.1)	0.81	**-3.3**	**(-5.2, -1.3)**	**0.007**	**34.4**	**(27.4, 41.3)**	**<0.001**
CD64 (MFI)^2^	**7,246.5**	**(5123.8, 9369.3)**	**<0.001**	**6,259.5**	**(4607.8, 7911.3)**	**<0.001**	**1,073.7**	**(779.4,1368.1)**	**<0.001**	**3,075.2**	**(2052.8,4097.7)**	**<0.001**
CD86 (MFI)^2^	250.5	(-955.4, 1456.3)	0.754	-982.7	(-3108.2, 1142.8)	0.491	**-3,375.4**	**(-5123.0,-1627.7)**	**0.002**	-304.0	(-902.8, 294.8)	0.451
CCR2 (MFI)^2^	16.4	(-217.83, 250.7)	0.907	45.6	(-114.8, 206.0)	0.689	**428.6**	**(237.3,620.0)**	**<0.001**	**959.1**	**(483.0,1435.3)**	**0.001**
HLA-DR (MFI)^2^	-950.6	(-2829.3, 928.1)	0.451	-6,444.4	(-12931.9, 43.2)	0.111	340.5	(-1815.3, 2496.3)	0.815	-440.8	(-1580.2, 698.5)	0.579
CD16 (MFI)^2^	-51.4	(-106.0, 3.1)	0.129	-83.6	(-252.7, 85.5)	0.457	**-1,366.0**	**(-1840.6,-891.5)**	**<0.001**	**-180.7**	**(-259.4,-101.9)**	**<0.001**
CD14 (MFI)^2^	-1,882.0	(-3799.5, 35.6)	0.116	309.4	(-2036.3, 2655.1)	0.836	8.5	(-355.6, 372.6)	0.972	**-1,806.3**	**(-2736.9,-875.6)**	**0.002**
TNF (MFI)^2^	-56.4	(-207.7, 95.0)	0.590	**-1,145.5**	**(-1793.5,-497.4)**	**0.004**	-196.4	(-409.6, 16.8)	0.137	-171.6	(-315.1, -28.1)	0.055
IL-6 (MFI)^2^	127.6	(4.4, 250.7)	0.097	62.2	(-83.2, 207.5)	0.536	69.3	(-40.9, 179.5)	0.335	109.3	(3.0, 215.6)	0.099
IL-1β (MFI)^2^	**-2,278.6**	**(-3446.5,-1110.7)**	**0.001**	**-4,800.3**	**(-7198.5,-2402.1)**	**0.001**	**-1,153.8**	**(-1796.9,-510.8)**	**0.003**	**-398.9**	**(-720.0,-77.8)**	**0.046**
Monocyte subsets gated using 5-receptor gating following LPS stimulation at 37 °C												
TNF (ΔMFI)^3^	-1,605.5	(-3525.2, 314.28)	0.184	**-6,897.6**	**(-11209.0,-2586.2)**	**0.009**	**-2,532.8**	**(-3233.9,-1831.6)**	**<0.001**	**-1,274.1**	**(-1644.7,-903.5)**	**<0.001**
IL-6 (ΔMFI)^3^	-339.5	(-823.4, 144.4)	0.273	-293.9	(-693.5, 105.7)	0.249	**-649.4**	**(-820.7,-478.1)**	**<0.001**	**-293.8**	**(-407.2,-180.4)**	**<0.001**
IL-1β (ΔMFI)^3^	**-9,152.4**	**(-13994.4,-4310.3)**	**0.002**	**-13,139.1**	**(-20722.1,-5556.1)**	**0.004**	**-3,894.4**	**(-5291.7,-2497.1)**	**<0.001**	**-3,030.2**	**(-3953.8,-2106.7)**	**<0.001**

^1^ Results of linear regression adjusted for age and sex. P-values from linear regression were adjusted using the Benjamini-Hochberg method. Adjusted p-values are shown.

^2^ Expression of phenotypic markers was assessed using geometric mean fluorescence intensity (MFI) on the indicated subset.

^3^ Data shown are the ΔMFI following subtraction of the MFI of the matched unstimulated sample incubated in parallel.

Bold values indicate statistically significant findings with adjusted p-value <0.05.

### CD14^low^CD16^-^ monocytes are phenotypically distinct and are associated with systemic inflammation

To interrogate the phenotype of this novel monocyte population, the surface expression of selected receptors was compared to other monocyte subsets. Despite these cells appearing adjacent to the Class subset based on CD14 and CD16 expression (see **Supplementary Figure 1E**), CD14^low^CD16^-^ monocytes exhibited significantly lower expression of CD163 and CD142 as compared to all other subsets and low expression of CD11b which was comparable to levels on NC monocytes (**Supplementary Figure 3**). In contrast, CD14^low^CD16^-^ monocytes exhibited the highest CXCR3 expression of all subsets. To determine whether the identified CD14^low^CD16^-^ subset contained cells with a dendritic cell (DC)-like phenotype, we further phenotyped a subset of acute COVID-19 samples with the DC markers CD1c, CD11c and CD209. The mean proportion of cells expressing either CD1c and/or CD209 was <6% for all monocyte subsets, suggesting minimal contamination with monocyte-derived or type 2 conventional dendritic cells (32–34). CD11c expression was highest on Int monocytes and lowest on the CD14^low^CD16^-^ subset, confirming the novel CD14^low^CD16^-^ subset is not enriched for cells with a conventional DC-like phenotype.

Given their reduced CD14 and CD16 expression, the CD14^low^CD16^-^ cells may be derived from traditional monocyte subsets following activation-mediated receptor shedding *in vivo*. To determine whether proportions of CD14^low^CD16^-^ monocytes were associated with systemic inflammation or viral burden, plasma levels of inflammatory and immune activation biomarkers and nasopharyngeal viral load were measured and indicated a significant positive association between CD14^low^CD16^-^ monocytes and TNF (p=0.006) and CXCL10 (p=0.016; **Supplementary Table 2**). Other biomarkers including those more indicative of myeloid cell activation and receptor shedding (ie soluble CD14 and CD163, CCL2) were not associated with this population, nor was the inflammatory factor CRP or nasopharyngeal viral load (**Supplementary Table 2**).

### The proportion of CD14^low^CD16^-^ monocytes is further increased following ex vivo culture

To investigate the inflammatory activity of monocytes in acute COVID-19, we evaluated intracellular cytokine levels in monocyte subsets *ex vivo* under both unstimulated and LPS-stimulated conditions. Given the well-documented potential for activated monocytes to shed CD14 and CD16 during *ex vivo* culture (35, 36), we initially evaluated methods for phenotyping monocyte subsets in COVID-19+ samples following resting of PBMCs *ex vivo*. Even in the absence of stimulation, *ex vivo* culture of cells for 4 h at 37 °C induced a significant expansion of CD14^low^CD16^-^ monocytes (mean difference vs un-cultured cells = +33.4%, p<0.001, **Figure 1H**) and a subsequent reduction in Class monocytes (-27.7%, p<0.001, **Figure 1E**) in individuals with acute COVID-19. The proportions of Int and NC subsets were also decreased in cultured cells as compared to cells analyzed immediately, but to a smaller extent (difference -5.3% and -1.4%, respectively, p<0.001 for both, **Figures 1F, G**). *Ex vivo* rested cells from control individuals exhibited a similar pattern of monocyte subset alterations, but to a substantially lower extent (**Figures 1E-H**). These data suggest that acute COVID-19 infection destabilizes monocyte surface phenotype which is further exacerbated upon *ex vivo* culture, impacting the ability to accurately characterize monocyte inflammatory status using traditional gating approaches.

**Figure 2:**
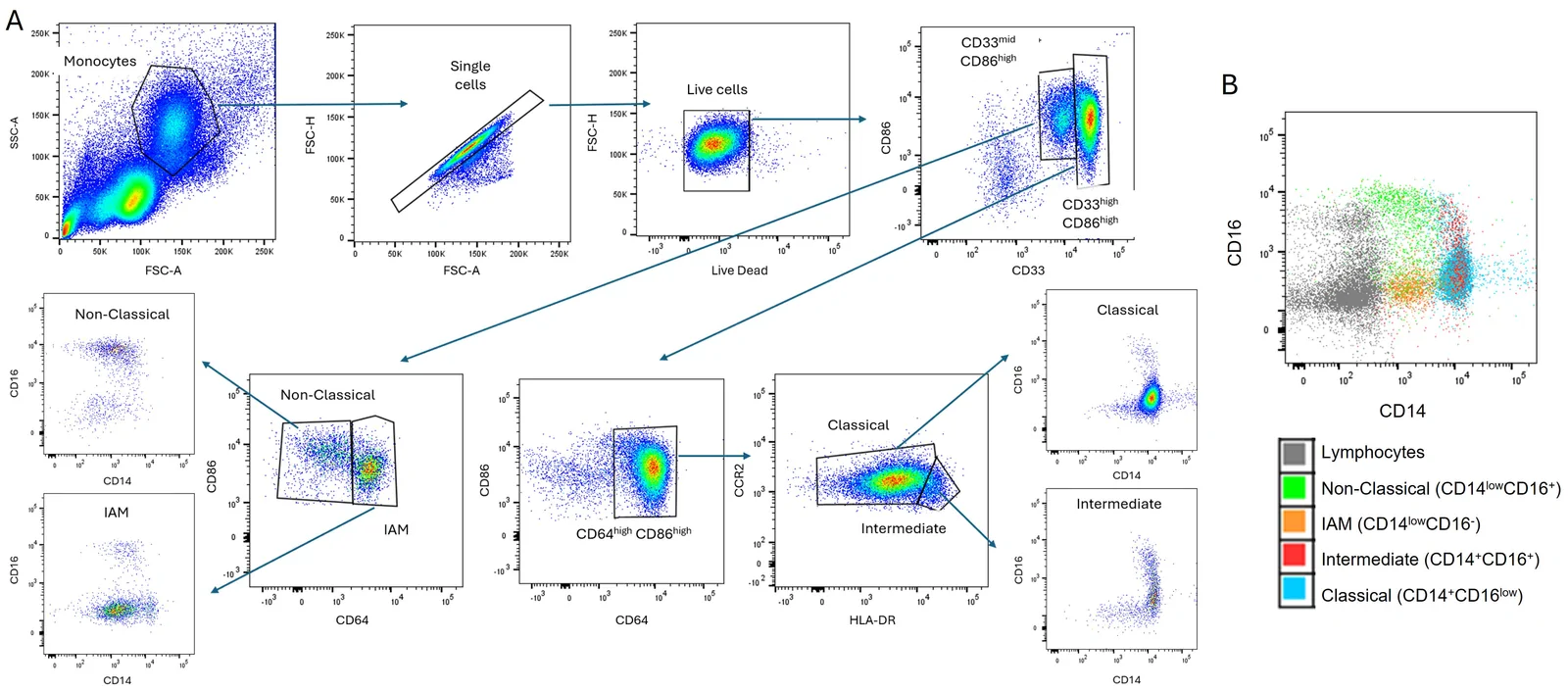
*Alternative gating of monocyte subsets using more robust surface markers.* **(A)** Gating of monocyte subsets based on surface expression of 5 receptors known to exhibit stable expression under stimulated settings. Monocyte subsets were defined as classical (CD33^high^CD86^high^CD64^high^CCR2^high^HLA-DR^mid^), intermediate (CD33^high^CD86^high^CD64^high^CCR2^mid^HLA-DR^high^), non-classical (CD33^mid^CD86^high^CD64^mid^) or infection-associated monocytes (IAM; CD33^mid^CD86^high^CD64^high^. **(B)** Overlay plot showing CD14 and CD16 expression on each of the indicated monocyte subsets, gated as shown in **(A)**, as compared to singlet lymphocytes (gated based on forward and side scatter).

We therefore utilized an alternative monocyte subset gating strategy optimized for cultured cells which avoids the measurement of unstable CD14 and CD16 markers (27). This strategy utilizes 5 receptors known to exhibit stable expression during stimulation and identifies monocyte subsets as classical (CD33^high^CD86^high^CD64^high^CCR2^high^HLA-DR^mid^), intermediate (CD33^high^CD86^high^CD64^high^CCR2^mid^HLA-DR^high^) and non-classical (CD33^mid^CD86^high^; see **Figure 2A**). When analyzing samples from individuals with acute COVID-19 infection, we identified an additional subset of monocytes with high CD64 expression (gated as CD33^mid^CD86^high^CD64^high^) which were distinct to CD33^mid^CD86^high^CD64^low^ NC monocytes (**Figure 2A**). As these cells were substantially more prevalent in COVID-19+ individuals, exhibited low but positive CD14 expression and were negative for CD16 staining (**Figure 2B**), they were thus considered to be analogous to the CD14^low^CD16^-^ subset defined above, and are hereafter referred to as infection-associated monocytes (IAM). We then compared the proportion of monocyte subsets identified using CD14/CD16 or the 5-receptor gating approach using unstimulated samples cultured *ex vivo* and found moderate but significant concordance between the proportions of Int monocytes (Spearman’s rho: 0.470, p<0.001), consistent with this subset representing a heterogenous population (9). However, high concordance between gating approaches was observed for all other subsets (rho: 0.712 – 0.919, p<0.001 for all, **Supplementary Figure 4**).

**Figure 3:**
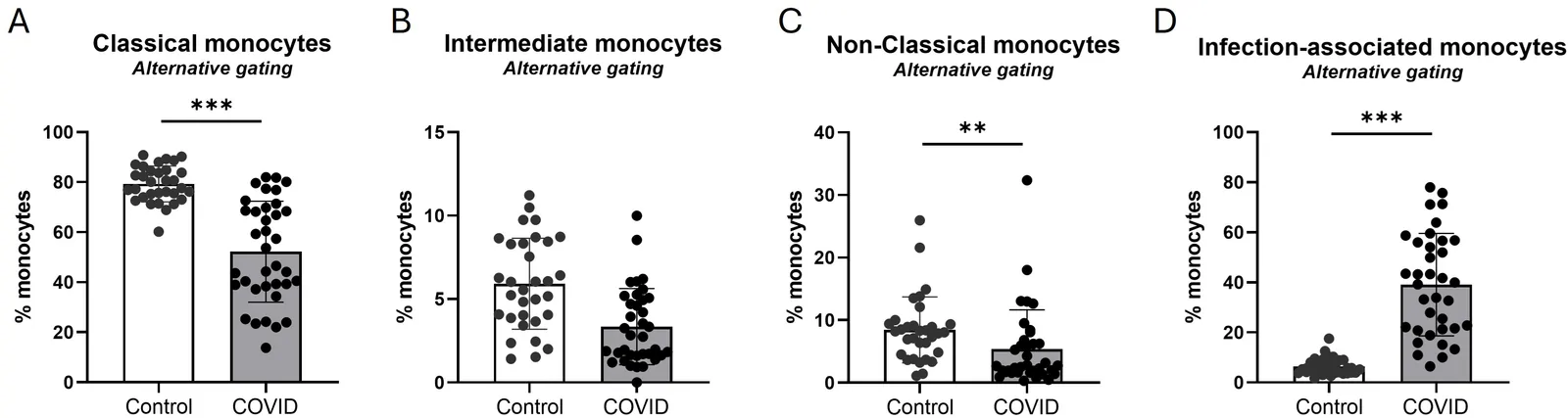
*Monocyte subset proportions in acute COVID-19 identified using alternative 5 receptor gating.* Proportions of monocyte subsets in samples cultured for 4 hours at 37 °C (without stimulation), **(A)** were identified using a robust 5 receptor-based gating and classified as Classical (CD33^high^CD86^high^CCR2^high^HLA-DR^mid^), Intermediate (CD33^high^CD86^high^CCR2^mid^HLA-DR^high^), **(B)** Non-Classical (CD33^mid^CD86^high^CD64^low^), **(C)** and Infection-associated monocytes (CD33^mid^CD86^high^CD64^high^), **(D)**. Graphs show mean and standard error of the mean for controls (unfilled bars) and COVID cases during acute infection (gray bars). Statistically significant differences between COVID cases and controls within each age group were determined by linear regression analysis adjusted for age and sex where *, ** and *** indicates p<0.05, <0.01 and <0.001, respectively.

Analysis of monocyte subset proportion using the 5-receptor gating approach confirmed a significant and substantial expansion of the CD14^low^CD16^-^ IAM population in *ex vivo* cells and a reduced proportion of other subsets which was statistically significant for Class and NC subsets (**Figure 3**). This parallel analyses thus confirmed the expansion of this novel IAM population using two independent gating strategies.

**Figure 4:**
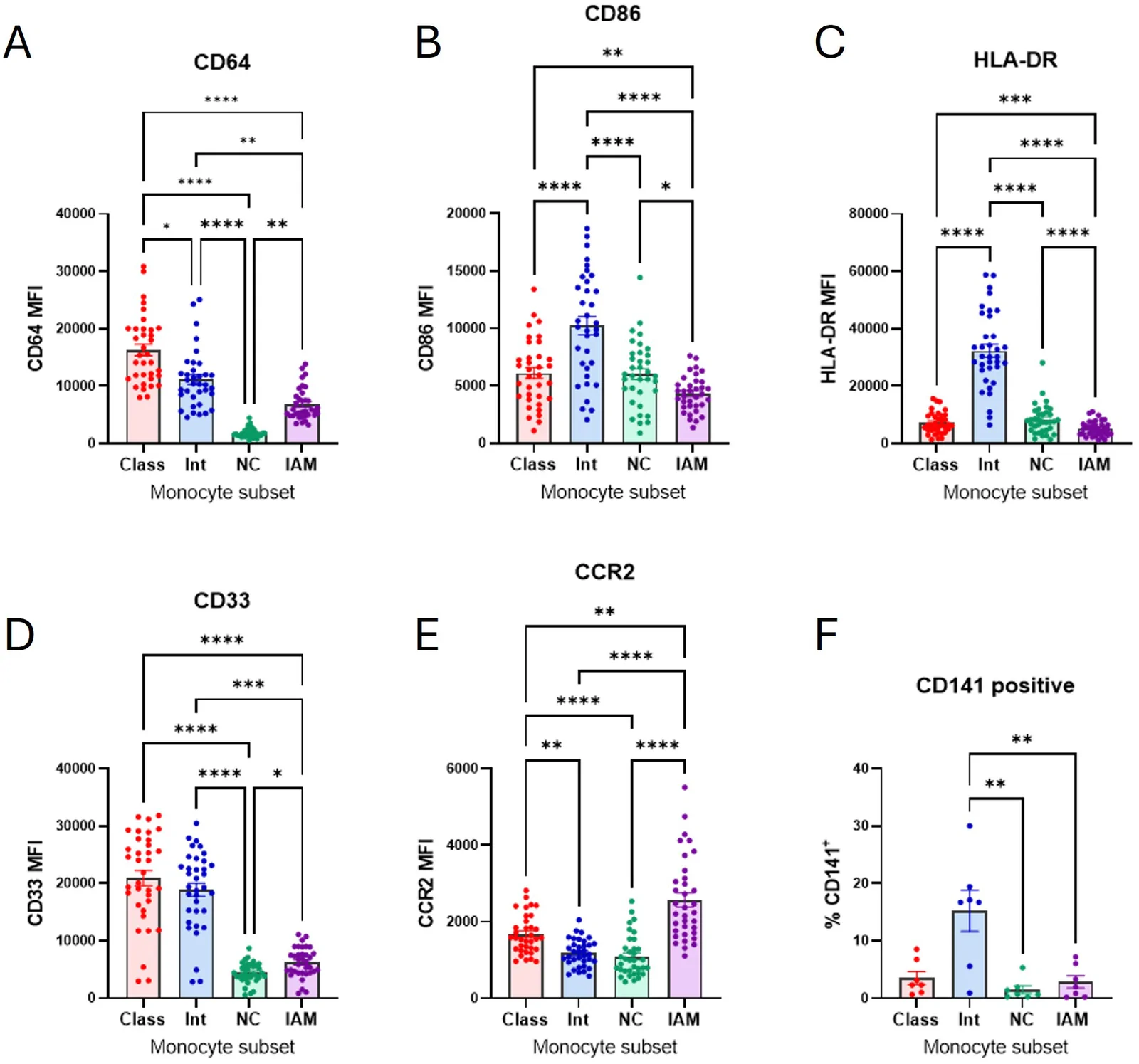
*Surface phenotype of novel IAM subset expanded in acute COVID-19 infection.* Surface immunophenotype of monocyte subsets from individuals with acute COVID-19 gating using the 5-receptor gating strategy as shown in **Figure 2**. Graphs show mean and standard error the mean of the median geometric mean fluorescence intensity (MFI) of the surface receptors CD64 **(A)**, CD86 **(B)**, HLA-DR **(C)**, CD33 **(D)**, CCR2 **(E)** and the percentage of cells in each subset which were positive for CD141 **(F)**. Statistically significant differences between subsets were determined using Friedman test and Dunn’s multiple comparison test where *, **, *** and **** indicates p<0.05, <0.01, <0.001 and <0.0001, respectively.

Phenotypic comparison of IAM suggested that while they were most similar to NC regarding reduced expression of CD64, CD86, HLA-DR and CD33 as compared to Class and Int subsets, IAM were distinct from NC monocytes as they exhibited significantly higher surface expression of CD64, CD33 and CCR2 but lower CD86 and HLA-DR expression (**Figure 4**). IAM exhibited the highest CCR2 expression of all subsets. Whilst a variable proportion of Int monocytes from individuals with acute COVID-19 were found to express CD141, expression of this marker was minimal in Class, NC and IAM populations, suggesting minimal contamination with cDC1 cells (**Figure 4**). In addition to monocyte subset alterations, acute COVID-19 was associated with significantly increased expression of CD64 on all monocyte subsets (adjusted p<0.001 for all, **Table 2**) which is consistent with previous reports in severe COVID-19 (37). The phenotype of NC and IAM monocytes were further altered in acute infection with increased CCR2 but decreased CD16 expression (adjusted p ≤ 0.001 for all) and decreased CD86 expression on NC (adjusted p=0.002) as compared to controls.

Taken together, these data indicate that acute COVID-19 infection is associated with expansion of a novel population of IAMs with a unique phenotype. Furthermore, monocyte perturbations induced by acute COVID-19 infection are further manifested *ex vivo* and include heightened destabilization of key monocyte receptors including CD14 and CD16.

### The inflammatory status of monocyte subsets is altered in acute COVID-19 infection

**Figure 5:**
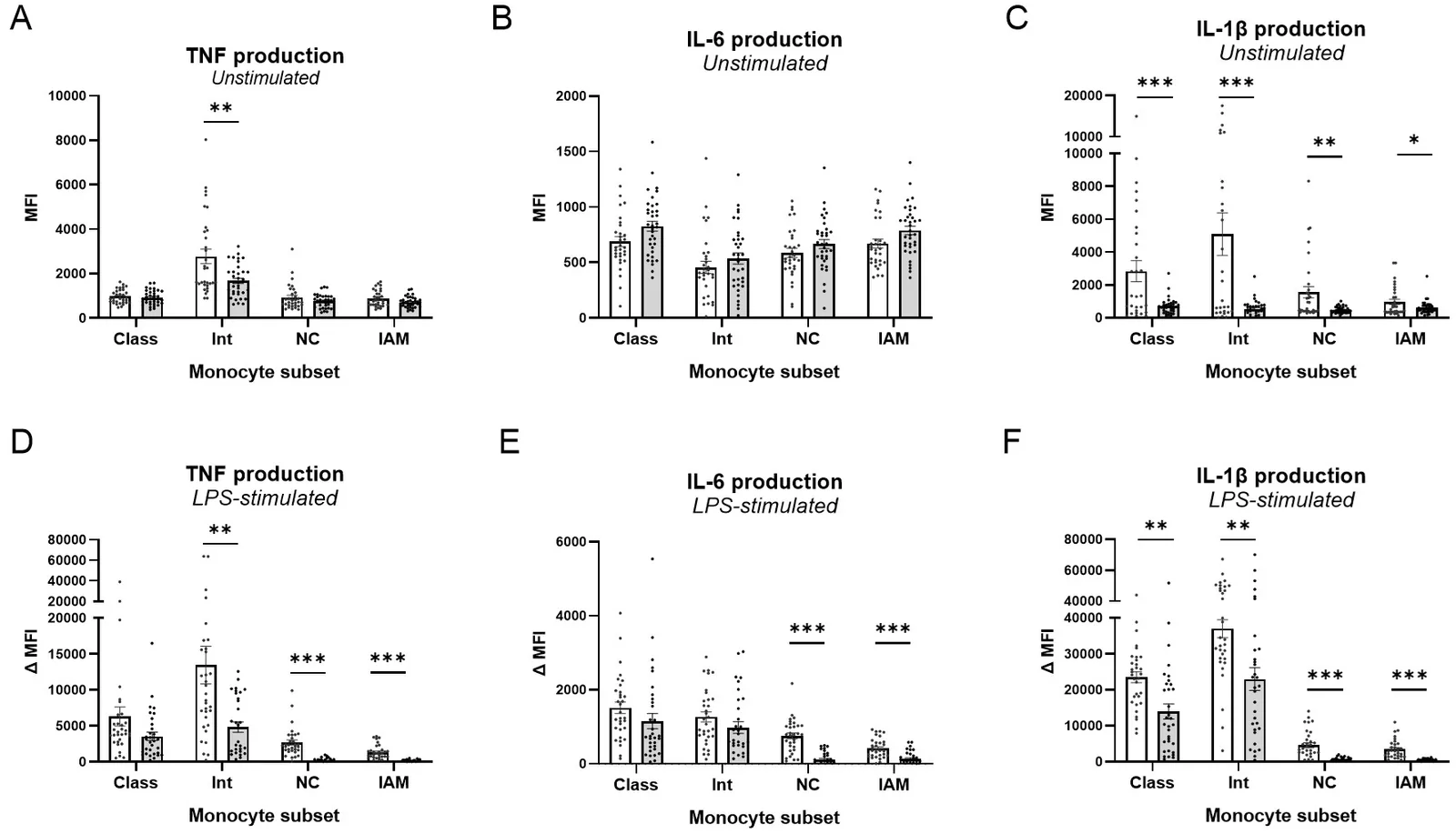
Inflammatory status of monocyte subsets in acute COVID-19. Intracellular levels of TNF **(A, D)**, IL-6 **(B, E)** and IL-1β **(C, F)** were evaluated in monocyte subsets identified using the 5 receptor-based gating approach in controls (unfilled bars) and individuals with acute COVID-19 (gray bars) following culture for 4 hours in the absence **(A–C)** or presence of LPS stimulation **(D–F)**. Data show geometric mean fluorescence intensity (MFI) and D-F represent the difference between stimulated and unstimulated conditions (Δ MFI). Graphs show mean and standard error of the mean. *, ** and *** indicate p<0.05, <0.01 and <0.001, respectively, as determined by linear regression analysis adjusted for age and sex.

The validated 5 receptor monocyte subset gating approach was then used to evaluate the impact of COVID-19 on monocyte inflammatory activity. In the absence of stimulation, monocytes cultured from acute COVID-19+ individuals contained significantly lower intracellular levels of IL-1β within all subsets as compared to controls (adjusted p<0.05 for all) while Int monocytes also contained significantly lower TNF levels (adjusted p=0.004, **Table 2**, **Figure 5**). Regarding the inflammatory response of monocytes to LPS stimulation, acute COVID-19+ individuals exhibited significantly lower LPS-stimulated production of IL-1β across all subsets, impaired TNF production in all subsets except Class monocytes, and impaired IL-6 production in NC and IAM subsets (**Table 2**, **Figure 5**).

We also evaluated the impact of acute COVID-19 infection on the polyfunctionality of monocyte responses. Whilst cytokine production was minimal in unstimulated monocytes, a slightly greater proportion of monocytes from controls contained 2 or more cytokines while COVID-19 cases showed a greater proportion of cells single positive for IL-6 but fewer IL-1β+ cells, consistent with the above findings (**Supplementary Figure 5A**). Following LPS stimulation, the polyfunctional cytokine responses in NC and IAM subsets were noticeably lower in acute COVID-19 infection as compared to controls, with a significant reduction in the proportion of cells producing 2 or 3 cytokines and conversely an increased proportion of cells exhibiting no inflammatory response to stimulation in both NC (62% expressing no cytokines vs 29% in controls) and IAM (63% vs 29% in controls; p<0.0001 for both, **Supplementary Figure 5B**).

Given NC and IAM monocytes exhibit lower expression of CD14 required for LPS signaling which could account for their impaired response to LPS stimulation, we evaluated whether these subsets also exhibited reduced inflammatory activity in response to CD14-indepentent toll-like receptor (TLR) stimulation. Following stimulation with both bacterial (Pam3CSK4, TLR1/2) and viral (R848, TLR7/8) TLR agonists, both NC and IAM subsets consistently produced lower levels of IL-1β, IL-6 and TNF as compared to both Class and Int subsets (**Supplementary Figure 6**), suggesting their impaired inflammatory activity was not agonist-specific. These data confirm acute COVID-19 infection is associated with a significantly impaired inflammatory activity of monocytes, particularly the NC and IAM subsets.

### Some COVID-19-related defects to monocyte phenotype and function persist for up to 3 months following acute infection

Previous reports indicate COVID-19-induced immunological changes can persist following resolution of acute infection (22), potentially contributing to ongoing immune dysfunction. To investigate this, we analyzed post-acute samples taken from individuals 1 and 3 months after diagnosis and compared them to control samples using regression analysis. These analyses revealed that proportions of Class and IAM subsets remained significantly lower and higher, respectively, in convalescent COVID-19+ individuals as compared to uninfected controls for at least 3 months post-acute infection (adjusted p ≤ 0.002 for all, **Table 3**). Heightened expression of CD16 persisted on IAM and Class monocytes 1 month (adjusted p<0.001 for both) but not at 3 months post-diagnosis. The IAM subset exhibited additional phenotypic differences in COVID-19+ as compared to control individuals, with decreased CD86 and CD14 but increased CCR2 expression persisting for at 3 months post-acute infection (**Table 3**).

**Table 3:** Persistent defects in monocytes 1 and 3 months post-acute COVID infection as compared to controls^1.^.

		1 month			3 months		
Parameter	Subset	D	95%	p (adjusted)	D	95%	p (adjusted)
Subset proportion (%)	Class	**-16.7**	**(-22.5,-10.9)**	**<0.001**	**-10.1**	**(-15.1,-5.0)**	**0.002**
	Int	-0.3	(-1.4, 0.8)	0.674	0.5	(-0.8, 1.9)	0.574
	NC	0.1	(-2.5, 2.6)	0.956	0.7	(-2.2, 3.6)	0.716
	IAM	**16.9**	**(11.4,22.5)**	**<0.001**	**8.8**	**(4.9,12.7)**	**<0.001**
CD64 (MFI)^2^	Class	371.4	(-811.3, 1554.1)	0.658	320.1	(-988.8, 1628.9)	0.716
	Int	673.8	(-182.8, 1530.4)	0.214	372.9	(-609.8, 1355.5)	0.585
	NC	161.5	(-23.7, 346.6)	0.164	170.5	(-34.4, 375.3)	0.187
	IAM	117.5	(-291.8, 526.8)	0.689	133.5	(-356.8, 623.8)	0.689
CD86 (MFI)^2^	Class	-378.1	(-1390.7, 634.5)	0.590	-321.9	(-1486.5, 842.7)	0.689
	Int	-1,786.2	(-3499.1, -73.3)	0.097	-1092.5	(-3189.1, 1004.2)	0.443
	NC	**-3,071.0**	**(-4769.9,-1372.2)**	**0.003**	-2067.5	(-4034.1, -100.9)	0.096
	IAM	**-1,256.0**	**(-1719.7,-792.2)**	**<0.001**	**-980.0**	**(-1491.3,-468.6)**	**0.002**
CCR2 (MFI)^2^	Class	241.6	(-98.6, 581.8)	0.267	155.3	(-160.5, 471.1)	0.461
	Int	68.8	(-119.5, 257.1)	0.596	28.5	(-182.6, 239.7)	0.834
	NC	274.7	(7.5, 541.9)	0.100	26.0	(-93.5, 145.5)	0.749
	IAM	**1,184.6**	**(649.5,1719.7)**	**<0.001**	**869.0**	**(396.1,1341.8)**	**0.003**
HLA-DR (MFI)^2^	Class	-518.8	(-2392.5, 1355.0)	0.689	-350.4	(-2815.9, 2115.0)	0.831
	Int	-2,984.2	(-9305.9, 3337.6)	0.481	-4145.6	(-11808.7, 3517.5)	0.419
	NC	-876.8	(-2618.9, 865.3)	0.451	-1039.5	(-2775.1, 696.1)	0.364
	IAM	-414.4	(-1604.4, 775.6)	0.620	-274.1	(-1556.7, 1008.5)	0.751
CD16 (MFI)^2^	Class	**-123.5**	**(-183.0,-64.0)**	**<0.001**	-89.0	(-161.6, -16.5)	0.051
	Int	-115.9	(-300.0, 68.2)	0.335	-17.6	(-238.0, 202.8)	0.900
	NC	-391.5	(-1075.7, 292.7)	0.388	253.6	(-667.8, 1175.0)	0.689
	IAM	**-208.4**	**(-299.5,-117.3)**	**<0.001**	-149.5	(-276.3, -22.8)	0.06
CD14 (MFI)^2^	Class	-1,527.0	(-3419.7, 365.8)	0.202	-1402.6	(-3573.4, 768.2)	0.321
	Int	-1,844.8	(-3480.5, -209.1)	0.072	-1871.9	(-3665.8, -78.1)	0.097
	NC	**-499.4**	**(-838.1,-160.7)**	**0.016**	-370.8	(-801.5, 59.97)	0.171
	IAM	**-2,521.2**	**(-3548.4,-1494.1)**	**<0.001**	**-2623.9**	**(-3797.0,-1450.9)**	**<0.001**
TNF (MFI)^2^	Class	-117.2	(-256.0, 21.6)	0.180	-179.5	(-341.8, -17.1)	0.077
	Int	**-958.8**	**(-1674.4,-243.1)**	**0.030**	**-1077.9**	**(-1930.3,-225.5)**	**0.044**
	NC	-253.0	(-477.9, -28.0)	0.073	-310.5	(-577.7, -43.3)	0.064
	IAM	**-205.0**	**(-352.1,-58.0)**	**0.023**	**-283.9**	**(-450.5,-117.3)**	**0.006**
IL-6 (MFI)^2^	Class	30.0	(-87.4, 147.3)	0.709	-11.3	(-151.6, 128.9)	0.900
	Int	2.4	(-142.5, 147.2)	0.980	-16.8	(-192.0, 158.5)	0.883
	NC	44.1	(-65.1, 153.2)	0.561	21.9	(-106.7, 150.5)	0.804
	IAM	31.9	(-70.1, 133.9)	0.658	8.5	(-112.2, 129.2)	0.907
IL-1b (MFI)^2^	Class	**-2,765.2**	**(-4132.6,-1397.7)**	**0.001**	**-2827.1**	**(-4503.3,-1151.0)**	**0.006**
	Int	**-5,384.2**	**(-8186.8,-2581.6)**	**0.002**	**-5278.7**	**(-8731.2,-1826.1)**	**0.013**
	NC	**-1,206.6**	**(-1958.6,-454.6)**	**0.009**	**-1242.7**	**(-2164.1,-321.2)**	**0.030**
	IAM	**-538.4**	**(-882.1,-194.6)**	**0.011**	**-662.9**	**(-1073.9,-252.0**	**0.009**
LPS stim TNF (ΔMFI)^3^	Class	-1,671.9	(-3952.4, 608.5)	0.250	-2698.1	(-5134.7, -261.5)	0.077
	Int	-4,939.5	(-10011.4, 132.4)	0.119	-4198.3	(-10487.8, 2091.2)	0.306
	NC	**-2,115.3**	**(-2938.4,-1292.2)**	**<0.001**	**-1934.0**	**(-2917.8,-950.2)**	**0.002**
	IAM	**-1,374.4**	**(-1816.7,-932)**	**<0.001**	**-1312.2**	**(-1841.2,-783.2)**	**<0.001**
LPS stim IL-6 (ΔMFI)^3^	Class	110.0	(-392.2, 612.1)	0.749	434.5	(-164.7, 1033.7)	0.256
	Int	114.4	(-295.6, 524.4)	0.689	548.0	(-12.4, 1108.4)	0.119
	NC	**-464.8**	**(-654.05,-275.6)**	**<0.001**	**-356.6**	**(-618.3,-94.9)**	**0.029**
	IAM	**-271.8**	**(-401.12,-142.5)**	**<0.001**	**-302.5**	**(-445.3,-159.6)**	**<0.001**
LPS stim IL-1b (ΔMFI)^3^	Class	5,912.4	(588.01, 11236.77)	0.076	3911.6	(-1230.0, 9053.2)	0.231
	Int	8,396.4	(841.0, 15951.9)	0.076	8122.3	(409.9, 15834.7)	0.096
	NC	**-3,138.2**	**(-4764.8,-1511.7)**	**0.002**	-2510.7	(-4661.4, -360.0)	0.063
	IAM	**-3,045.8**	**(-4141.0,-1950.6)**	**<0.001**	**-2854.7**	**(-4182.1,-1527.2)**	**<0.001**

^1^ Results of linear regression, adjusted for age and sex. P-values from linear regression were adjusted using the Benjamini-Hochberg method. Adjusted p-values are shown. Values shown represent differences in the geometric mean fluorescence intensity (MFI) of each parameter on the indicated subset as compared to uninfected controls.

^2^ Expression of phenotypic markers was assessed using geometric mean fluorescence intensity (MFI) on the indicated subset identified using 5 receptor gating.

^3^ Data for LPS-stimulated samples show the ΔMFI following subtraction of the MFI of the matched unstimulated sample incubated in parallel.

Bold values indicate statistically significant findings with adjusted p-value <0.05.

Regarding the inflammatory activity of monocytes, levels of IL-1β in unstimulated cells from COVID-19+ individuals remained lower in all monocyte subsets at both 1 and 3-month convalescent timepoints as compared to controls (adjusted p<0.05 for all). Reduced levels of TNF in unstimulated Int and IAM subsets were also not reversed during convalescence. Whilst impaired responses to *ex vivo* stimulation observed in acute infection were fully resolved in Class and Int subsets at convalescent timepoints, this was not the case for NC and IAM monocytes which exhibited persistently impaired inflammatory responses up to 3 months post infection. Together, these data indicate that many COVID-19-induced perturbations to monocyte phenotype and function including impaired inflammatory activity persist for up to 3 months after infection.

### COVID-19 infection induces persistent T and NK cell activation

To evaluate persistent immune dysfunction in other immune cell types, we assessed T and NK cell activation in acute and convalescent samples (gating strategy shown in **Supplementary Figure 6**). The proportion of CD4+ and CD8+ T cells and NK cells expressing the early activation marker CD69 were significantly increased in acute COVID-19 infection (adjusted p<0.001 for all as compared to uninfected controls, **Table 4**) while proportions of HLA-DR+CD38+ cells (typically considered markers of chronic activation) were not significantly altered. NK cells in acute infection also exhibited significantly increased expression of CD38 (adjusted p<0.001) and a lower proportion of CD56^bright^ NK cells (adjusted p=0.005). Proportions of activated CD69+ T and NK cells remained significantly elevated as compared to uninfected controls up to 3 months post infection. Taken together, these data suggest that COVID-19 has a significant impact on the phenotype and function of both innate and adaptive immune cells which persists for at least 3 months following acute infection.

**Table 4:** Differences in T and NK cell activation in COVID+ individuals at acute and convalescent time points as compared to uninfected controls^1,2.^.

	Acute infection			1 month post-diagnosis			3 months post-diagnosis		
	Difference	95% CI	p (adjusted)	Difference	95% CI	p (adjusted)	Difference	95% CI	p (adjusted)
CD4+ T cells									
CD69+	**11.7**	**(8.8,14.6)**	**<0.001**	**12.8**	**(8.3,17.2)**	**<0.001**	**14.1**	**(9.4,18.8)**	**<0.001**
HLA-DR+/CD38+	0.9	(-0.9, 2.8)	0.451	-0.3	(-1.9, 1.3)	0.754	-1.4	(-3.2, 0.3)	0.202
CD8+ T cells									
CD69+	**11.7**	**(7.4,15.9)**	**<0.001**	**11.5**	**(5.9,17.2)**	**0.001**	**13.4**	**(7.2,19.6)**	**<0.001**
HLA-DR+/CD38+	2.6	(-4.0, 9.3)	0.569	1.6	(-5.9, 9.3)	0.751	-3.5	(-11.8, 4.8)	0.541
NK cells									
CD69+	**18.4**	**(11.9,24.9)**	**<0.001**	**11.3**	**(5.2,17.4)**	**0.003**	**9.9**	**(2.3,17.6)**	**0.039**
HLA-DR+/CD38+	4.6	(-0.5, 9.6)	0.143	3.3	(-2.7, 9.2)	0.410	-1.3	(-7.4, 4.9)	0.757
CD56bright	**-6.7**	**(-10.5,-2.8)**	**0.005**	-5.1	(-9.7, -0.5)	0.076	-6.1	(-11.6, -0.6)	0.077
CD38 MFI^3^	**12,266.9**	**(7835.2,16698.7)**	**<0.001**	2,516.9	(-916.6, 5950.4)	0.250	1,212.7	(-1931.8, 4357.3)	0.580

^1^ Results of linear regression, adjusted for age and sex. P-values from linear regression were adjusted using the Benjamini-Hochberg method. Adjusted p-values are shown.

^2^ Data show percent positive for the indicated marker unless stated otherwise.

^3^ Indicates geometric mean fluorescence intensity (MFI) on the indicated subset.

Bold values indicate statistically significant findings with adjusted p-value <0.05.

## Discussion

This study investigated the impacts of acute COVID-19 infection on monocyte activity utilizing a robust immunophenotyping gating strategy that facilitated identification of a novel population of CD14^low^CD16^-^ monocytes that were expanded in acute COVID-19 infection. This monocyte population exhibited an inert inflammatory phenotype which was refractory to *ex vivo* stimulation and analysis of longitudinal samples indicated the persistence of this population for 3 months after infection, potentially impacting the ability of monocytes to respond to further immune challenge *in vivo*. These findings add new information to the accumulating evidence that COVID-19 induces an enduring reshaping of immune phenotype and function and indicate that this can occur in individuals of a range of ages and disease severity.

Acute COVID-19 infection has been consistently associated with reduced proportions of Class monocytes, as observed in the present study, but varying changes are reported regarding impacts on NC and Int subsets (12–18). Here we demonstrate a substantial destabilization of the CD14 and CD16 surface receptors traditionally used for monocyte subset phenotyping in COVID-19+ individuals, which may contribute to the variation observed in previous studies, particularly for NC and Int subsets which can be challenging to identify with high purity (38). Here we identified an expanded, novel subset of CD14^low^CD16^-^ monocytes with an inert phenotype in COVID-19 which was validated using two parallel phenotyping approaches, one using traditional CD14/CD16 gating and the other utilizing monocyte markers which exhibit more consistent expression under stimulation and disease settings (27). Despite low expression of CD14 and CD16, these cells morphologically and phenotypically resemble monocytes in that they exhibit high forward and side scatter, are negative for CD3/CD19/CD56, and express monocyte receptors including CD33, CD86, CD64, CCR2, CD11b and HLA-DR. Expression of DC markers including CD1c, CD209 and CD141 was minimal to absent on these CD14^low^CD16^-^ cells, making it unlikely that they represent a subset of conventional or monocyte-derived DC, while their expression of CD33 and CD11b is not consistent with these cells representing a type of plasmacytoid DC (32–34).

In light of this, the inert IAM population identified here is unique in that it has not been widely reported under homeostatic or pathogenic settings, which may be due to a failure to capture these cells using traditional CD14/CD16 profiling. Even under non-pathogenic settings, monocyte heterogeneity extends beyond the traditional Class, Int and NC subtype designations (39) and in older individuals, a population of ‘immature’ CD14^low^CD16^-^ monocytes potentially analogous to the population identified here is expanded (3). A novel population of CD127+, hypo-inflammatory monocytes has been identified in COVID-19 infection and also in individuals with rheumatoid arthritis (40), supporting the existence of novel monocyte subsets with specialized functions under certain pathogenic conditions. The source of the expanded IAM observed here remains unclear, but our observation that this population further increased after *ex vivo* culture may suggest an enhanced susceptibility of monocytes from COVID-19+ individuals to ADAM17-mediated CD14 shedding (36). However, IAM were phenotypically distinct to Class monocytes regarding expression of receptors other than CD14, including higher expression of CCR2 and CXCR3, potentially implicating recent bone marrow mobilization and/or enhanced migration potential. Cryopreservation of the samples utilized here may have potentiated the pre-existing fragility of surface receptors, suggesting whole blood analyses are required to further investigate this population. However, the observation that *ex vivo* increases in the IAM proportion were significantly more prevalent in COVID-19+ samples suggests an inherent difference in monocyte phenotype and/or stability was present prior to cryopreservation. The reduced CD14 and CD16 expression on IAM may reflect a population of monocytes which have undergone activation-induced alterations *in vivo* to dampen monocyte responses and prevent further inflammation. Indeed, the significant association with *in vivo* levels of inflammatory markers and the increased proportion of CD14^low^CD16^-^ monocytes in individuals with severe infection in consistent with this. In this regard, the IAM population may be analogous to CD56-negative NK cells which are expanded in chronic HIV and hepatitis C virus infection (41). The expansion and role of IAM in COVID-19 and potentially other viral infections warrants further investigation.

Here we observed a phenotype of impaired basal and stimulated inflammatory responses in monocytes in acute COVID-19 which was most prominent in NC and IAM subsets. Impaired cytokine production by monocytes has been previously reported in severe acute COVID-19 (26) but here we note this also in our cohort which included more mild disease presentations. We also observed that while some monocyte perturbations induced during acute COVID-19 infection were quickly reversed, others including the expansion of inert monocytes persisted for at least 3 months post-acute infection. This is consistent with other longitudinal studies that report a slow restoration of monocyte phenotype following acute COVID-19 infection, with changes to some parameters such as classical monocyte proportion only stabilizing after 5 months (22). The endurance of these changes within short-lived monocytes is consistent with observations of monocyte reprogramming or ‘training’ following severe (23, 26) and even moderate (24) COVID-19 infection. This reprogramming is characterized by an altered transcriptional profile and epigenetic changes including modified chromatin accessibility in convalescent individuals (23, 24, 26, 42). Furthermore, evidence of this reprogramming has been detected in hematopoietic stem cell precursors 1 year after infection and linked to IL-6 stimulation during acute infection (43). Some (26, 43) but not other (42) studies associate this monocyte reprogramming with hyper inflammatory responses to stimulation but in our cohort we observed the converse. These varied presentations suggest that while monocyte reprogramming may be common in convalescent COVID-19 infection, heterogeneity exists regarding the nature and functional implications of these changes. Whether these enduring immune changes observed post-acute infection may contribute to the pathogenesis of long COVID-19 symptoms in some individuals remains to be evaluated, however, a recent report of persistent downregulation of inflammatory genes including IL-1β in classical monocytes from individuals with long COVID (44) suggests persistent monocyte dysfunction may be clinically relevant in at least some individuals. Whilst not the focus on this study, we also observed persistent activation of T and NK cells 3 months post-infection, suggesting ongoing and widespread immune dysfunction may persist post-acute COVID-19 infection.

This observational study was limited to a small number of participants and was not designed to evaluate associations between observed phenotypic changes and clinical outcomes. Many monocyte parameters analyzed here including subset proportion are known to be altered by age and sex, so a strength of this study is that analyses were adjusted for these parameters. This study was performed on cryopreserved PBMC which are typically more inert to *ex vivo* stimulation than fresh whole blood samples (45), so it would be of significant interest to extend these findings using whole blood analyses as well as include evaluation of total cell numbers in addition to population proportions.

In conclusion, through using a novel monocyte phenotyping approach better suited to pathogenic settings, this study has identified expansion and persistence of an inert population of monocytes following COVID-19 infection. This approach can be applied to other disease settings to determine whether this novel monocyte population is unique to COVID-19 or a common but poorly documented characteristic of monocyte responses to acute infection.

## Data Availability

The raw data supporting the conclusions of this article will be made available by the authors, without undue reservation.
