# Supplementary material for: Induction of dysfunctional CD14lowCD16- monocytes in acute COVID-19 infection
Source: Front Immunol. 2026 Aug 25;17:1814210. doi: 10.3389/fimmu.2026.1814210 (PMC13550204; doi:10.3389/fimmu.2026.1814210)
Supplement: Supplementary file 1 [file DataSheet1.pdf]

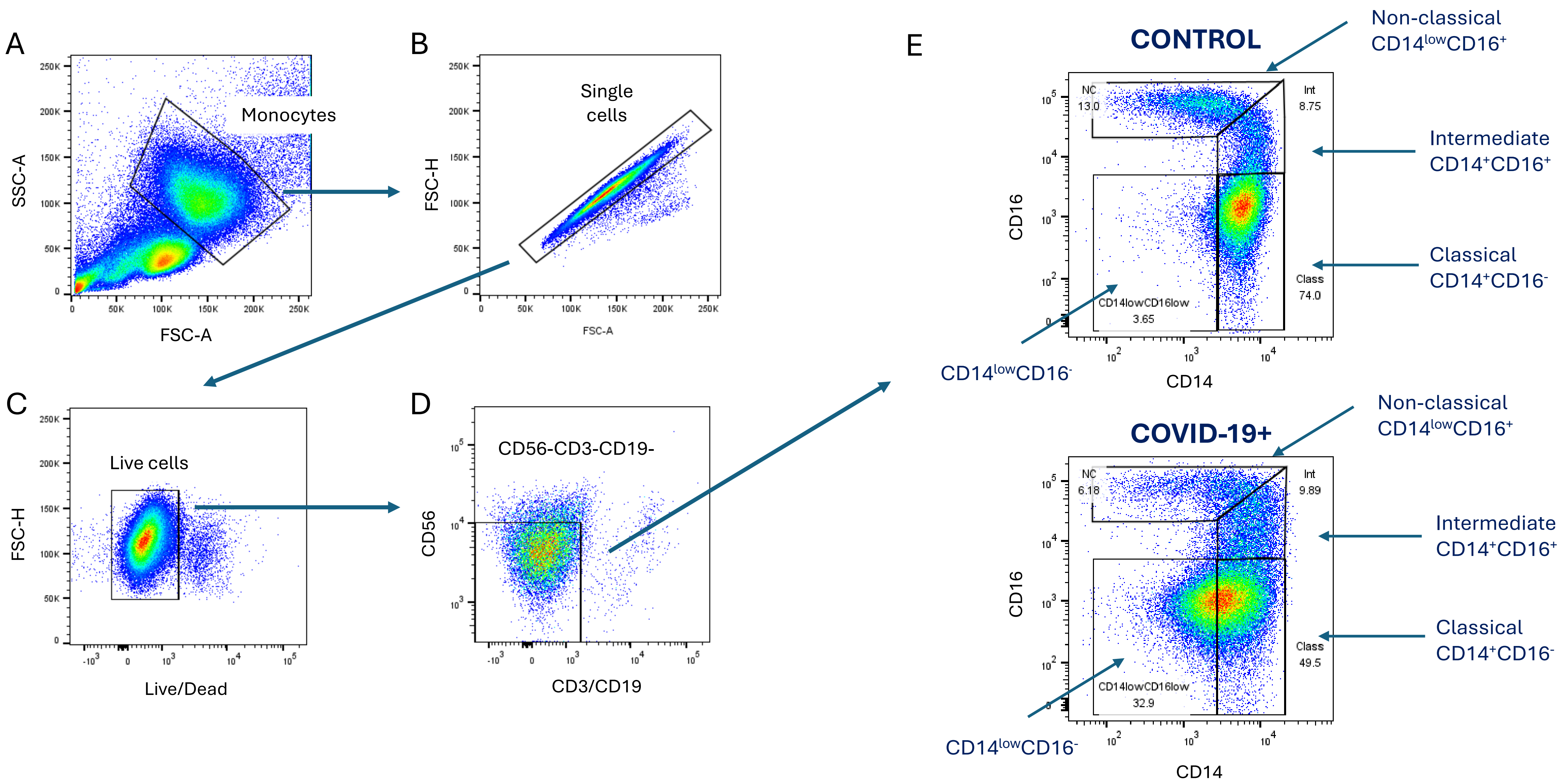

Supplementary Figure 1: *Gating of monocyte subsets based on surface expression of CD14 and CD16.*
Monocytes were gated based on size and granularity (A), singlets (B) and live cells (C) which lacked surface expression of CD56/3/19 (D). Monocyte subsets were identified based surface expression of CD14 and CD16 (E) and classified as classical (Class; CD14+CD16-), Intermediate (Int; CD14+ CD16+) and Non-classical (NC; $CD14^{low}CD16+$). An additional gate of $CD14^{low}CD16^{-}$ cells was applied to capture populations expanded in COVID-19 cases. E shows an example plot from an uninfected control (upper panel) and an acute COVID-19+ case (lower plot).

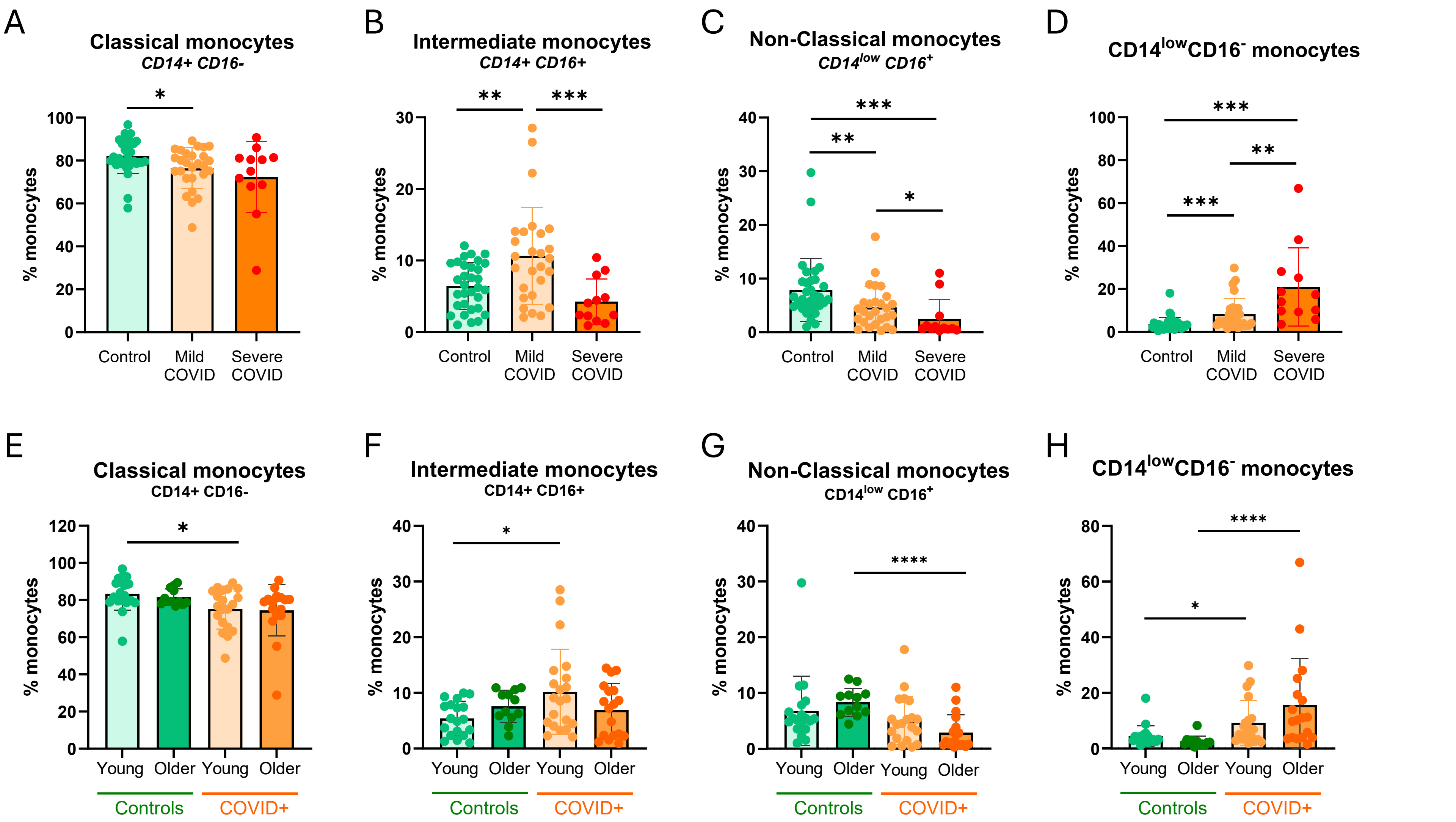

Supplementary Figure 2: *Impact of disease severity and age on expansion of novel $CD14^{low}CD16^{-}$ monocyte subsets in acute COVID-19*

The proportion of classical (CD14+CD16-; A&E), intermediate (CD14+CD16+; B&F), non-classical ($CD14^{low}CD16+$; C&G) and $CD14^{low}CD16^{-}$ (D&H) monocytes was determined using CD14/CD16 gating in PBMC immediately following defrosting. Upper graphs (A-D) show data for uninfected controls (green) or individuals with mild (non-hospitalised, light orange) or severe (hospitalised, dark orange) COVID-19 infection. Bottom graphs (E-H) show data for young (<40 years) and older (>60 years) uninfected controls (green bars) or COVID-19+ individuals during acute infection (orange bars). Graphs show mean and standard error of the mean. *, ** and *** indicate p<0.05, <0.01 and <0.001 as determined by Mann-Whitney U test.

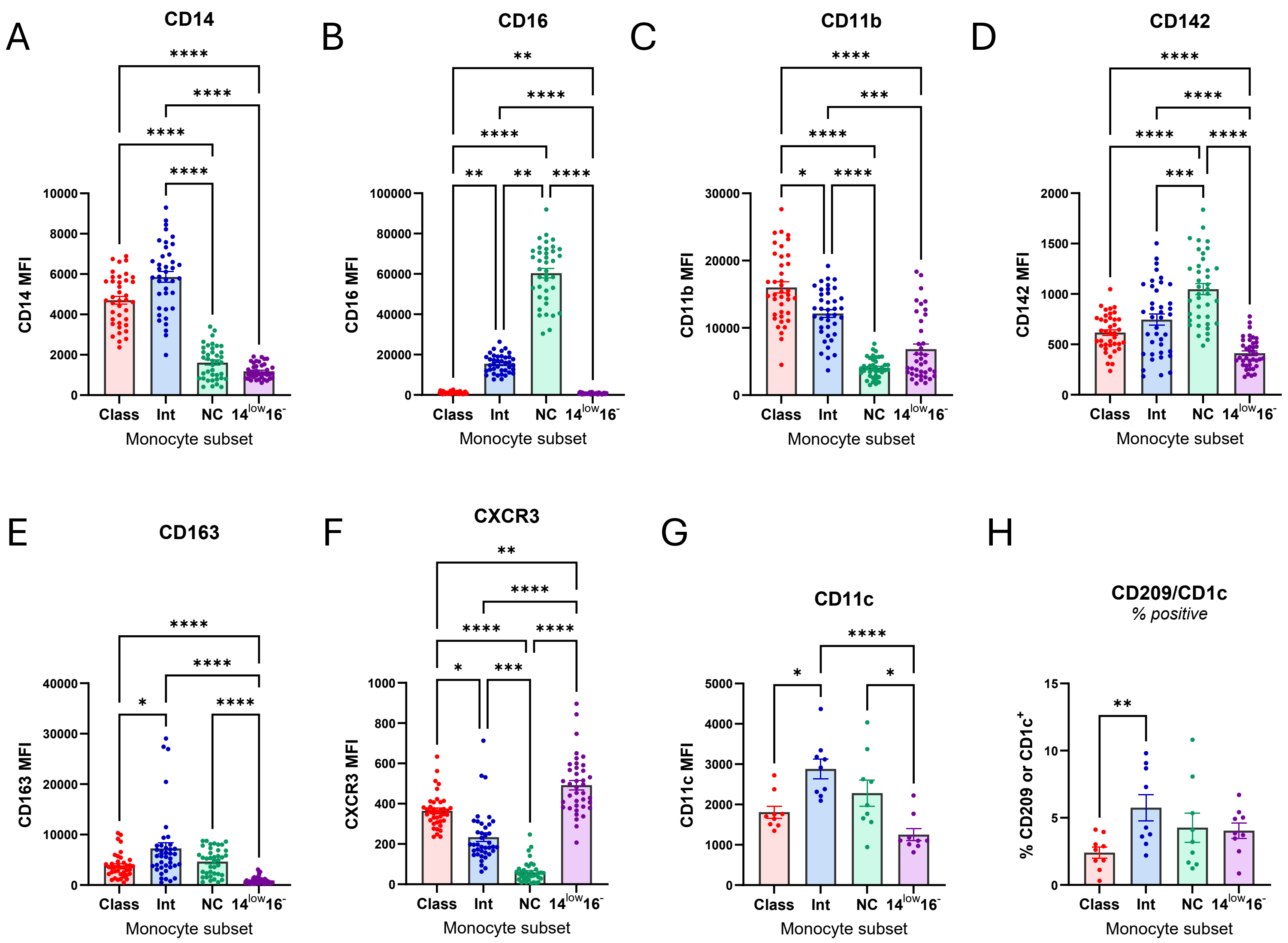

Supplementary Figure 3: *Surface phenotype of novel monocytes expanded in acute COVID-19 infection.*

Surface immunophenotype of monocyte subsets from individuals with acute COVID-19 identified using CD14/CD16 gating as shown in Supplementary Figure 1. Graphs show mean and standard error the mean of the median geometric mean fluorescence intensity of the surface receptors CD14 (A), CD16 (B), CD11b (C), CD142 (Tissue factor, D), CD163 (E), CXCR3 (F), CD11c (G) or the percentage of cells within each subset which expressed either CD209 and/or CD1c (H). Statistically significant differences between subsets were determined using Friedman test and Dunn's multiple comparison test where *, **, *** and **** indicates p<0.05, <0.01, <0.001 and <0.0001, respectively.

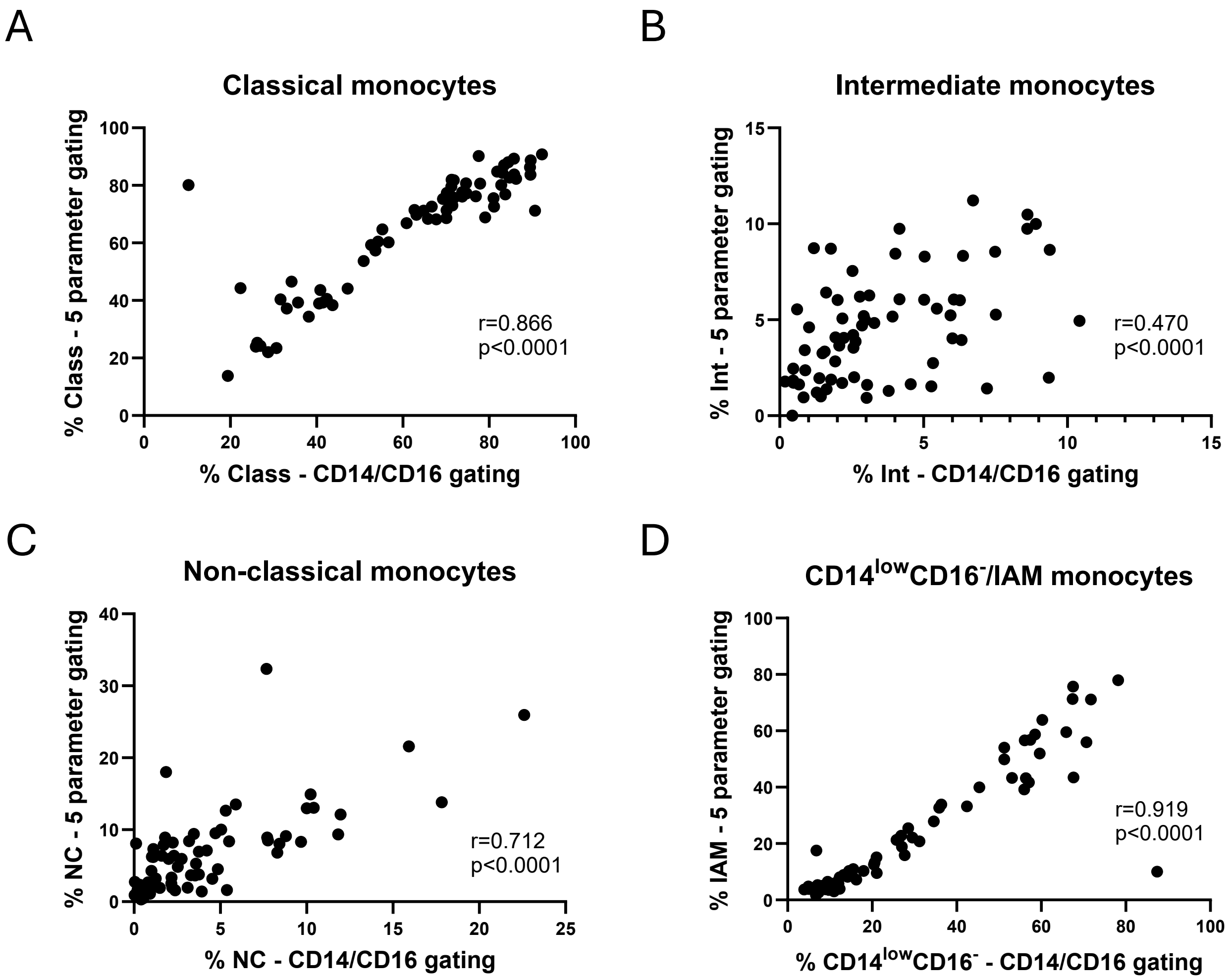

Supplementary Figure 4: *Correlation between monocyte proportions gated with traditional CD14/CD16 or an alternative 5-receptor gating strategy.*
Monocytes were gated using traditional gating strategies as shown in Supplementary Figure 1 or an alternative 5-receptor gating strategy as shown in Figure 2. Graphs show the correlation between proportions of monocyte subsets identified using both approaches for A) Classical, B) Intermediate, C) non-classical and D) $CD14^{low}CD16^{-}$/IAM monocyte subsets. Significant associations assessed using Spearman's rho.

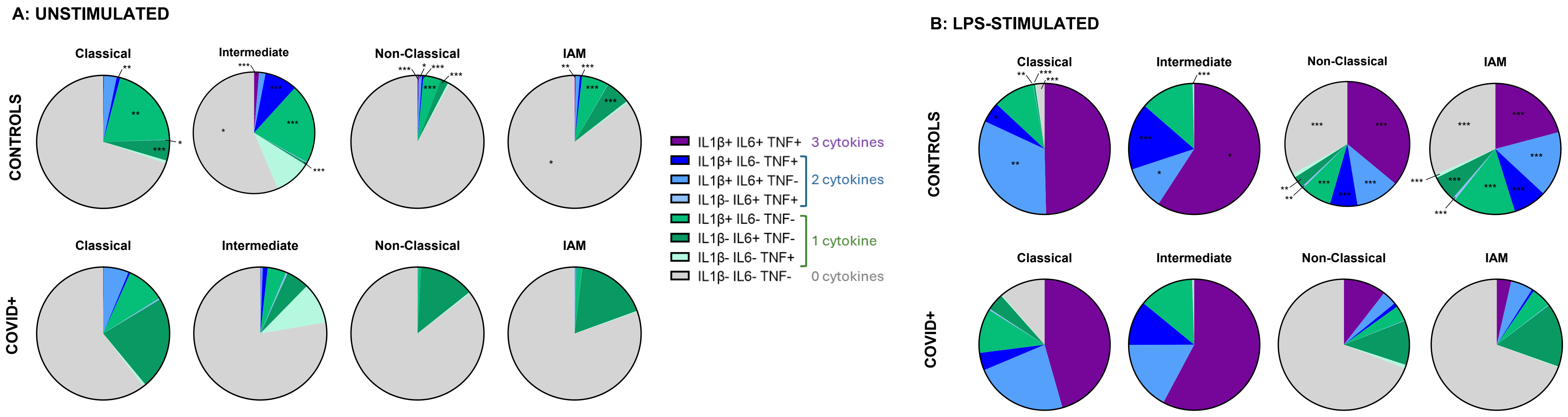

Supplementary Figure 5: *Basal and stimulated inflammatory cytokine production by monocyte subsets in acute COVID infection*

The proportion of monocytes within the indicated subsets which were positive for intracellular TNF, IL-6 and IL-1β was assessed by immunophenotyping in controls (top rows) and acute COVID Infection (bottom rows) following culture at 37°C for 4 hours in either the absence (A) or presence (B) of LPS. Charts show the mean proportion of monocytes within each subset which were positive for 0 (grey wedges), 1 (green wedges), 2 (blue wedges) or 3 (purple wedges) cytokines, as indicated in the legend. Statistically significant differences between controls and COVID-19 cases for each cell sub-population were evaluated using Mann-Whitney U test where *, ** and *** indicate $p<0.05$, 0.01 and 0.001 respectively (indicated in the upper graphs for each panel).

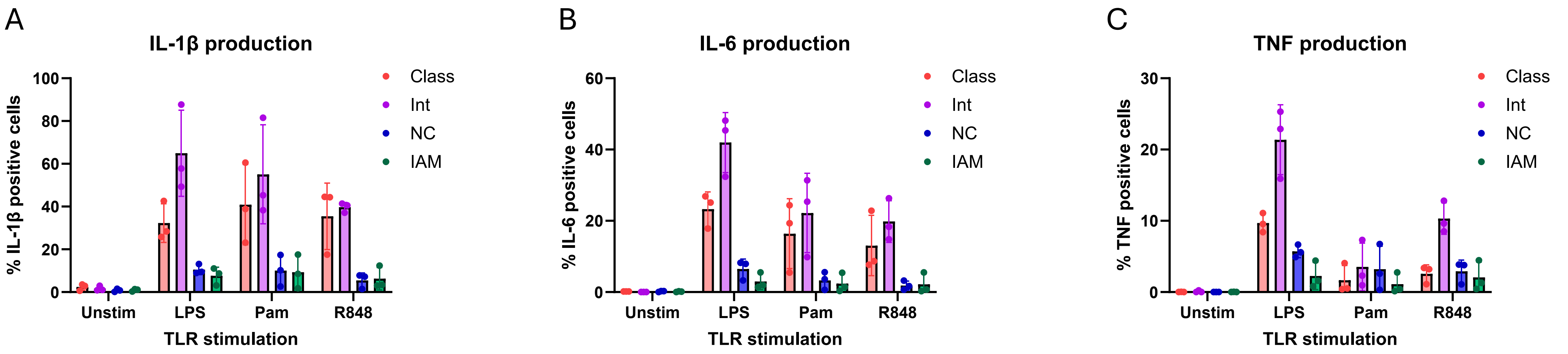

Supplementary Figure 6: *Inflammatory cytokine production by monocyte subsets in response to TLR agonists.*
Intracellular levels of IL-1β (A), IL-6 (B) and TNFα (C) were evaluated in monocyte subsets identified using the 5 receptor-based gating approach in healthy controls after incubation for 4 hours in the absence (Unstim) or presence toll-like receptor agonists lipopolysaccharide (LPS, TLR4 agonist; 50ng/mL), Pam3CSK4 (Pam; TLR1/2 agonists; 500ng/mL) or R848 (TLR7/8 agonist; 1µg/mL). Data are shown for classical (Class), intermediate (Int), non-classical (NC) and infection-induced monocytes (IAM). Graphs show mean and standard error of the mean of the percentage of cytokine positive monocytes. Statistical analyses not performed due to low replicate numbers.

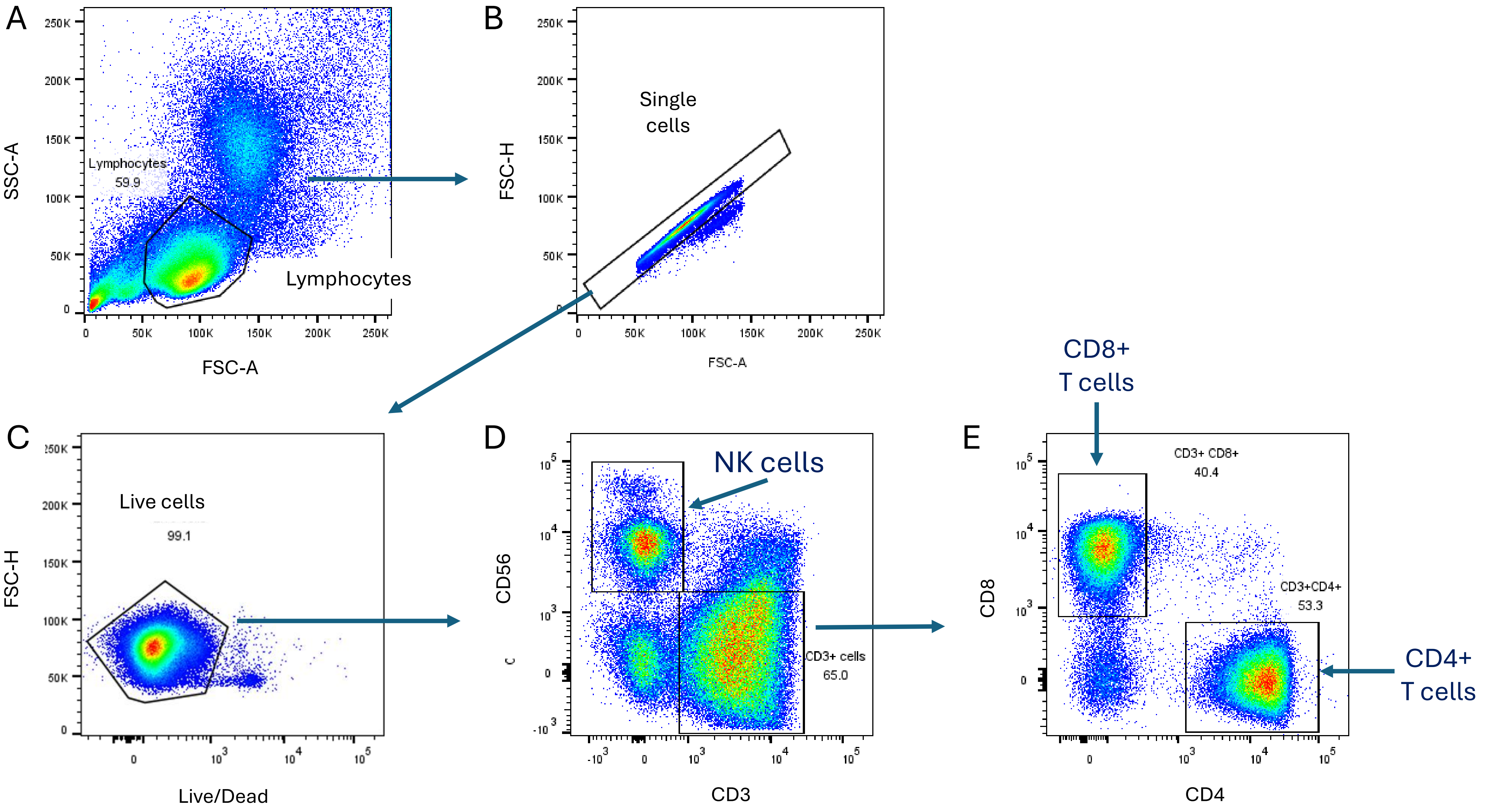

Supplementary Figure 7: *Gating strategy for identification of T and NK cells.*
Lymphocytes were gated based on size and granularity (A), singlets (B) and live cells (C). NK cells were gated as CD3-CD56+ cells (D) and CD3+ T cells were subsequently gated into CD4+ and CD8+ (E) subsets.
