# Supplementary material for: Induction of dysfunctional CD14lowCD16- monocytes in acute COVID-19 infection
Source: Front Immunol. 2026 Aug 25;17:1814210. doi: 10.3389/fimmu.2026.1814210 (PMC13550204; doi:10.3389/fimmu.2026.1814210)
Supplement: Supplementary file 2 [file Table1.docx]

**Supplementary Table 1:** Details of antibodies used in immunophenotyping analyses

| **Antibody and conjugate** | **Supplier** | **Catalogue #** | **Clone** |
| --- | --- | --- | --- |
| CCR2 PE | R&D | FAB151P | 48607 |
| CD1c BB515 | BD | 565054 | F10 |
| CD11b FITC | BioLegend | 301330 | ICRF44 |
| CD11c PE | BD | 340713 | SHCL-3 |
| CD141 BB515 | BD | 1A4 | 565084 |
| CD14 APC | BD | 555399 | M5E2 |
| CD14 BV650 | BD | 563419 | M5E2 |
| CD142 BV421 | BD | 744003 | HTF-1 |
| CD16 BUV495 | BD | 612944 | 3G8 |
| CD16 PE Cy7 | BD | 557744 | 3G8 |
| CD163 BV786 | BD | 741003 | GH/61 |
| CD19 APC Cy7 | BD | 560727 | HIB19 |
| CD3 APC Cy7 | BioL | 557832 | SK7 |
| CD3 PerCp Cy5.5 | BD | 340949 | SK7 |
| CD33 BV421 | BD | 562854 | WM53 |
| CD38 PE | BD | 347687 | HB7 |
| CD4 BV421 | BioLegend | 344632 | SK3 |
| CD56 AF700 | BD | 557919 | B159 |
| CD56 APC | Beckman Coulter | IM2474 | N901 |
| CD64 PerCp 5.5 | BioLegend | 305024 | 10.1 |
| CD69 Pe Cy7 | BD | 557745 | FN50 |
| CD8 APC Cy7 | BD | 557834 | SK1 |
| CD86 BUV737 | BD | 612784 | 2331 FUN-1 |
| HLA-DR BV711 | BioLegend | 307644 | L243 |
| HLA-DR PerCP Cy5.5 | BioLegend | 307630 | L243 |
| IL-1β AF647 | BD | 567773 | AS10 |
| IL-6 FITC | BD | 554696 | MQ2-6A3 |
| TNF BV785 | BioLegend | 502948 | MAb11 |

**Supplementary Table 2:** Change in soluble parameters associated with a 1% increase in CD14^low^CD16^low^ monocytes among COVID cases at acute infection^1^.

| **Soluble parameter** | **estimate** | **95% CI** | **p-value (unadjusted)** |
| --- | --- | --- | --- |
| CRP (ug/mL) | 0.04 | -0.15,0.23 | 0.650 |
| CD14 (ng/mL) | 3.97 | -12.73,20.67 | 0.644 |
| CCL2 (pg/mL) | 6.62 | -1.43,14.67 | 0.115 |
| CXCL10 (pg/mL) | 7.65 | 1.67,13.63 | 0.016 |
| CD163 (ng/mL) | 3.96 | -8.6,16.52 | 0.540 |
| TNF (ng/mL) | 0.27 | 0.09,0.46 | 0.006 |
| Viral load^2^ | -575,680 | -1868379,717018 | 0.388 |

^1^ Results of linear regression, adjusted for age and sex.

^2^ Nasopharyngeal viral load at diagnosis, expressed as copies of SARS CoV2 N1 detected in 1mL of nasopharyngeal swab eluate media.
