# Supplementary material for: Induction of dysfunctional CD14lowCD16- monocytes in acute COVID-19 infection
Source: Front Immunol. 2026 Aug 25;17:1814210. doi: 10.3389/fimmu.2026.1814210 (PMC13550204; doi:10.3389/fimmu.2026.1814210)
Supplement: Supplementary file 3 [file Table2.docx]

**SUPPLEMENTARY METHODS**

SUPPLEMENTARY METHODS 1: Model equation to assess changes among cases and controls

$$Y_{ij}=\beta_{0}+\beta_{1}X_{i}+\beta_{2}\text{age}_{i}+\beta_{3}\text{sex}_{i}$$

$Y_{ij} =$ Immunological parameter value for individual *i* at timepoint *j*

$X_{i}=$ 0 if individual i is a control, 1 if individual *i* is a case

$\text{age}_{i}=$ age in years of individual *i*

$\text{sex}_{i} =$sex of individual *i*

$\beta_{0}$ = population average in controls at age reference and sex reference

$\beta_{1} =$mean difference in outcome for a case relative to a control of the same age and sex.

We fit three models for timepoints *j* = acute, one month post infection and three months post infection. We reported the estimated value of $\beta_{1}$ and the 95% confidence interval.

SUPPLEMENTARY METHODS 2: Model equation to assess differences in soluble parameter values associated with increases in CD14^low^CD16^low^

$$Y_{i}=\beta_{0}+\beta_{1}\text{CD14}^{low}\text{CD}{16}_{i}^{low}+\beta_{2}\text{age}_{i}+\beta_{3}\text{sex}_{i}$$

$Y_{i} =$soluble factor value for individual *i* at acute infection

$\text{CD14}^{low}\text{CD}{16}_{i}^{low}=\text{CD14}^{\text{low}}CD{16}^{\text{low}}$ value for individual *i* at acute infection

$\text{age}_{i}=$ age in years of individual *i*

$\text{sex}_{i} =$sex of individual *i*

$\beta_{1} =$mean increase in soluble factor value associated with a 1% increase in $\text{CD14}^{\text{low}}CD{16}^{\text{low}}$
